# Investigating and Multi-Objective Optimizing WEDM Parameters for Al6061/Mg/MoS_2_ Composites Using BBD and NSGA-II

**DOI:** 10.3390/ma17235894

**Published:** 2024-12-01

**Authors:** Vagheesan Senthilkumar, Anbazhagan Nagadeepan, K. K. Ilavenil

**Affiliations:** 1 Department of Mechanical Engineering, SRM TRP Engineering College, Trichy 621105, Tamilnadu, India; trpvsk12@gmail.com; 2 Department of Chemistry, School of Engineering and Technology, Dhanalakshmi Srinivasan University, Trichy 621112, Tamilnadu, India; ilavenilkk.set@dsuniversity.ac.in

**Keywords:** WEDM, Al-Mg-MoS_2_ composites, Material Removal Rate (MRR), surface roughness (SR)

## Abstract

This study aims to optimize the Wire Electrical Discharge Machining (EDM) process parameters for aluminum 6061 alloy reinforced with Mg and MoS_2_ using the Box–Behnken (BBD) design and the non-dominated sorting genetic (NSGA-II) algorithm. The objective is to enhance the machining efficiency and quality of the composite material. The Box–Behnken (BBD) design was utilized to design a set of experiments with varying levels of process parameters, comprising pulse-on time, servo volt, and current. The material removal rate and surface roughness were considered as machining responses for optimization. These responses were measured and used to develop a mathematical model. The NSGA-II, a multi-objective optimization algorithm, was then applied to search for the optimal combination of process parameters that simultaneously maximizes the material removal rate and minimizes the electrode wear rate and surface roughness. The algorithm generated and evolved a set of Pareto-optimal solutions, providing a trade-off between conflicting objectives. The results of the optimization process were analyzed to identify the optimal process parameters that lead to improved machining performance. The study revealed optimal Wire Electrical Discharge Machining (WEDM) parameters for Al6061/Mg/MoS_2_ composites using NSGA-II. The optimized parameters, including a pulse-on time (Ton) of 105 µs, servo voltage (SV) of 35 V, and peak current (PC) of 31 A, resulted in a Material Removal Rate (MRR) of 7.51 mm^3^/min and a surface roughness (SR) of 1.97 µm. This represents a 15% improvement in the MRR and a 20% reduction in the SR compared to non-optimized settings, demonstrating the efficiency of the BBD-NSGA-II approach.

## 1. Introduction

Aluminum and its alloys are extensively used in the aerospace, automotive, and electronic industries due to their exceptional strength-to-weight ratio, corrosion resistance, and high thermal conductivity [1,2,3,4,5]. In order to enhance its stiffness, wear, specific strength, fatigue, and creep-fatigue properties, aluminum is reinforced with a metal, ceramic, or organic compound that is added to mend the base metal properties and thereby offer a higher flexibility in adapting the properties to engineering applications [6]. The possible choices to strengthen the metal/alloy matrix include reinforcing elements such as Al_2_O_3_, SiC, B, B4C, BN, and AlN [7,8,9]. Al6061 composites, reinforced with materials like Mg and MoS_2_, offer superior tribological and mechanical properties, which are essential for lightweight yet durable components in aircraft and automotive parts. Compared to Mg-based composites, aluminum composites provide better corrosion resistance, while they are more cost-effective and lightweight compared to Cu-based composites. However, aluminum composites can be less thermally stable than Zn-based counterparts, which is a consideration in high-temperature applications. Studies such as those in [10,11] provide a comprehensive overview of these trade-offs. These materials have seen increasing applications in potential automotive industries and many other industries, including aerospace, the military, and electronic packaging. There are numerous manufacturing techniques that can be used to fabricate AMMCs, including friction stir casting, pressure less infiltration, and powder metallurgy [12,13]. Stir casting is an established and reasonable method that can create products with complex shapes using a variety of materials and processing conditions and is the most cost-effective way to produce AMMCs. Stir casting costs between one-third and one-half as much as other, more expensive, approaches used to fabricate composite materials.

Traditional machining techniques have limitations such as being unable to precisely cut intricate designs, a low energy efficiency, limited recycling, etc. Nonconventional machining techniques like EDM, ECM, AWJM, and LBM are alternatives that get around these limitations [14,15]. Aeronautical structures, hydraulic and injection mold parts, ejection dies, and form tools are just a few of the intricate forms that WEDM can fabricate [16]. In WEDM, the work and wire materials (tools) interact thermoelectrically to conduct the machining. High-hardness materials may be readily machined with WEDM, since there is no direct contact between the tool and the work material, requiring just a basic clamping system [17]. When utilizing a WEDM to machine aluminum and other AMMCs, the performance is primarily affected by a number of variables, including the off-and-on pulse time, current, dielectric flow, feed (wire), voltage (gap), etc. According to a literature review and recent studies [18], some investigational work has been carried out and optimization tools have been used to optimize the surface finish, properties (mechanical and morphological), and material removal rate of stir-casted Al-MMCs during wire EDM. These studies, however, have some practical limitations due to the absence of multi-objective optimization tools that can resolve complex problems with multiple, competing responses. Because of the unpredictable nature of WEDM and also the additional complexity caused by reinforcements to the aluminum matrix material, suitable and ample analytical models for the prognosis of machine response characteristics are lacking. Consequently, WEDM has to be experimentally investigated utilizing samples of MMC based on aluminum, which requires the further development of predictive regression models because of the impact of the machining parameters on the process and the unpredictability of the processes.

In order to obtain the best performance, this research has to be used. Manufacturing businesses employ the multi-objective optimization tool NSGA-II to achieve maximum removal (MRR) and minimum surface variation (roughness) [19,20,21].

It is essential to highlight this study’s contributions, emphasizing its novelty, impact, and unique approach. By applying the Box–Behnken design together with NSGA-II to optimize WEDM performance on Al6061/Mg/MoS_2_, the composites of this study offer a fresh perspective with high prediction accuracy and low error rates.

The current effort is to optimize the multi-objective criteria in which the weights have been allocated to different performance metrics to fulfil the needs of diverse sectors, with the goal of addressing these issues. The findings of this research study will be able to provide researchers and practitioners with new recommendations for understanding the effects of different factors on the responses to wirecut EDM of Al/Mg/MoS_2_ hybrid MMCs.

## 2. Materials and Methods

**Figure 1:**
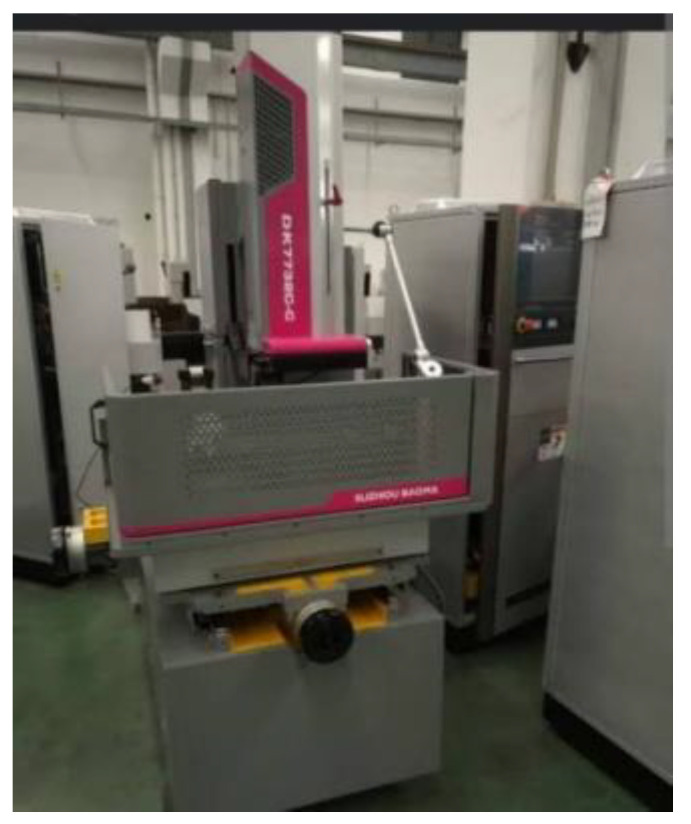
DK-7732 wirecutter/discharger machine.

**Figure 2:**
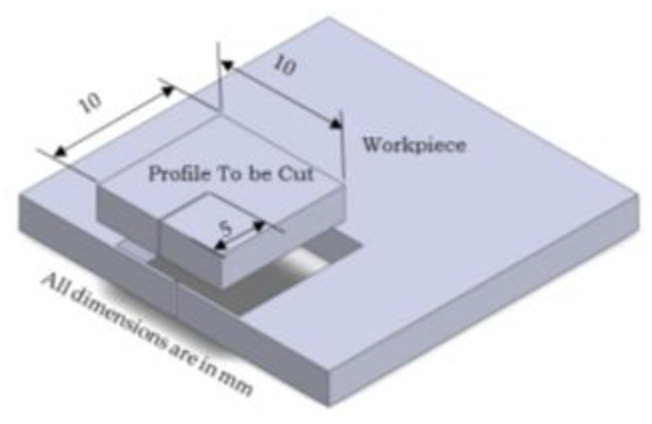
Profile.

The study’s matrix material was pure aluminum (Al6061), with a purity of 99.5 percent and particle size of 44 µm; on the other hand, the magnesium weight percentage was kept constant at 4% and the molybdenum weight percentage was altered with different weight percentages (2, 4, and 6) to prepare the 3 samples A, B, and C. The powders were first uniformly combined using ball-milling equipment, which was performed in an argon gas environment with the addition of toluene to remove oxidation. The furnace was also heated to 120 °C for 90 min to remove any inconsistent components from the powder mixture. To eliminate any last traces of moisture, the powder mixture was heated in a furnace. Finally, green compaction was carried out using a uniaxial hydraulic machine with a compaction pressure of 600 MPa, a sintering temperature of 500 °C, and a sintering period of 120 min. The completed product had the dimensions of 50 mm × 50 mm × 10 mm and was eventually reduced to 5 mm for wirecut EDM machining. On the AMMC Al-mg-MoS_2_ with a 5 mm thickness. Figure 1 shows the CNC wirecut EDM DK7732 with 3 kVA (power) and a BMW (3000) controller manufactured by Suzhou Baoma Numerical Control Equipment Co., Ltd. (Suzhou, China) and the cut profile was shown in Figure 2.

The Box–Behnken experiment strategy [22], which is a statistical experimental design method that enables the exploration of the process parameter space efficiently and offers a balanced and limited set of experimental points, was utilized for three different samples (A, B, and C) for the interactions between the input and output parameters in order to investigate the composite materials’ machinability properties. Deionized water was utilized to wash away the waste materials produced during the machining process, and a Φ 0.25 mm brass wire coated with zinc was employed as the electrode for cutting. The selection of input parameters, like the peak (pc) current, pulse-on (Ton) time, and servo (sv) voltage is critical in determining the surface (SR) roughness and material removal (MRR) rate. Table 1 shows the selection of input parameters, which were determined by prior pilot tests and an in-depth examination of the existing literature. The present study investigated the response characteristics of the Material Removal Rate (MRR) and surface roughness (SR), in which the surface roughness was tested using the surftest SJ-210, under the specified conditions viz. a travel distance of 4 mm and speed of 0.6 mm/s. After cleaning the surftest SJ-210 using acetone, the roughness tester was employed to assess the surface roughness (SR) at three distinct points, and subsequently the mean of the three values was chosen. The MRR was determined by using Equation (1) subsequent to the measurement of machining time using a stopwatch [23,24].

(1) $$MRR=\frac{L\;H}{t}$$

where:

*L*—length of cut;
*H*—thickness of work material; and
*t*—time of machining (in mins).

Table 2 shows Box–Behnken design with response values 

## 3. Results and Discussion

### 3.1. ANOVA (Multivariant Analysis)

The BBD and experimental results of the machining capabilities of the WEDM process are shown in Table 3. The backward elimination procedure excludes unnecessary factors, and the “*p*-value” must be less than or equal to 0.05 for the study to be considered significant at a 5% significance level (α = 0.05). Under ideal circumstances, the model’s ability to predict the correlation coefficient R’s ideal response value has been assessed. The best area to look at the reactions under investigation was found using regression analysis, which fits mathematical models to the experimental data.

Using the Design Expert software V13.0, the data from the experiment runs were evaluated and developed. Table 3 shows the importance of each coefficient for the MRR and SR. A higher significance level corresponds to a stronger degree of correlation between the observed and anticipated values [25]. All of the samples had “R-squared” values higher than 0.92, indicating a substantial correlation between the values of the expected and experimental responses. Additionally, the “Adj R-squared” values are closer to the R-squared values, which are logically compatible with the results of the current analysis. Furthermore, all of the samples had enough precision values greater than 4, which suggests a suitable indication and allows for the use of the current model to navigate the design space. The standard deviations for the MRR and SR across the samples were as follows: Sample A had an MRR standard deviation of 0.142 mm^3^/min and an SR standard deviation of 0.063 µm; Sample B had an MRR standard deviation of 0.207 mm^3^/min and an SR standard deviation of 0.140 µm; and Sample C had an MRR standard deviation of 0.176 mm^3^/min and an SR standard deviation of 0.172 µm. These standard deviations provide valuable insight into the measurement variability and precision, further strengthening the credibility and reliability of the experimental results.

ANOVA was used to analyze the data, and Table 4, Table 5 and Table 6 present the significance of the experimental results for different models together with the corresponding *p*-values. There were shown to be three significant linear correlation coefficients (A, B, and C), three interaction coefficients (AB, AC, and BC) and three quadratic coefficients (A^2^, B^2^, and C^2^). To highlight how the variables interact, the *p*-values of each model term were examined. The *p*-values for all the models were smaller than 0.05, indicating that they were very significant. The lack-of-fit F value > 0.05 indicates that the model was not significant because of the relative pure error. In general, unless the model is sufficiently fit to confirm its applicability, the analysis and optimization of the fitted response surface may yield inadequate or wrong findings. The *p*-value is used to evaluate each coefficient’s significance as well as the degree of dependency between the variables. Effect sizes under 0.05 are regarded as significant.

The data from the BBD were fitted using an experimental relationship for the MRR and SR and are represented by the equation of a II-order polynomial with interface terms, and the final results in coded factors from Equations (2) to (7) are as follows:

Response equations for Sample A:

(2) $$MRR=+3.75-0.0867*A-0.0167*B-0.0092*C+0.0115*A*B+0.0010*A*C-0.0010*B*C-0.0187*A^{2}-0.0037*B^{2}-0.0117*C^{2}$$

(3) $$Ra=+7.28-0.0688*A+0.0688*B+0.0175*C-0.0200*A*B-0.0025*A*C-0.0075*B*C-0.2310*A^{2}+0.0490*B^{2}+0.0615*C^{2}$$

Response equations for Sample B:

(4) $$MRR=+3.81-0.0868*A-0.0167*B-0.0093*C+0.0165*A*B+0.0060*A*C+0.0040*B*C-0.0152*A^{2}-0.0002*B^{2}-0.0182*C^{2}$$

(5) $$Ra=+6.60-0.0938*A+0.0562*B+0.0300*C+0.0050*A*B-0.0275*A*C-0.0075*B*C-0.2160*A^{2}+0.0890B^{2}+0.0515*C^{2}$$

Response equations for Sample C:

(6) $$MRR=+3.61-0.0818*A-0.0167*B-0.0092*C+0.0065*A*B-0.0040*A*C-0.0060*B*C-0.0152*A^{2}-0.0102*B^{2}-0.0082*C^{2}$$

(7) $$Ra=+6.92-0.0771*A+0.0771*B+0.0258*C-0.0033*A*B-0.0358*A*C+0.0258*B*C-0.2177*A^{2}+0.0623*B^{2}+0.0582*C^{2}$$

### 3.2. NSGA-II Multi-Objective Optimization

The sophisticated multi-objective optimization method NSGA-II employs non-dominated sorting to identify optimum solutions that meet many objectives simultaneously. NSGA-II is a well-established multi-objective optimization algorithm recognized for its efficiency in complex problems, leveraging a fast non-dominated sorting approach, an efficient crowded distance estimation, and a straightforward crowded comparison operator [26]. Kodali et al. [27] utilized NSGA-II to address a grinding machining operation involving dual objectives, four constraints, and ten decision variables. NSGA-II has since been widely adopted for optimizing WEDM processes in various materials, including Ti 6–2-4–2 alloy [28], AISI 5160 steel [29], high-speed steel [30], and AISI D3 tool steel [31,32]. The MRR and Ra for WEDM parameters, which rely on Ton, V, and A, are maximized and minimized. The MRR and Ra conflict, and therefore optimizing one may degrade the other, which can be addressed using NSGA-II by non-dominated selection to find non-dominated solutions. The crowd comparing operator and crowd distance rank the non-dominated level to pick it. These strategies preserve the population variety and avoid an algorithm that converge to a local optimum.

The NSGA-II technique consists in the following steps:

i. To start, initialize the population (N) using the maximum and lowest input parameters.
ii. Then, determine fitness functions for each individual, incorporating the MRR and surface roughness.
iii. Sort the original population non-dominatedly.
iv. Elect individuals based on crowding space and ranking, and then generate offspring via cross-over and mutation procedures with 0.95 and 0.01 factors, respectively.
v. Combine the populaces of the paternities and offspring and rank and crowd the next generation.
vi. Stop if 500 generations have been reached; otherwise, move to Step 4.

Thus, maximize MRR = minimize (-MRR) = f (Ton, SV, PC) and minimize Ra = f (Ton, V, A).

The above is influenced by the following:

100 ≤ Ton ≤ 110 µs; 30 ≤ SV ≤ 40 V; 30 ≤ PC ≤ 32 A.

**Figure 3:**
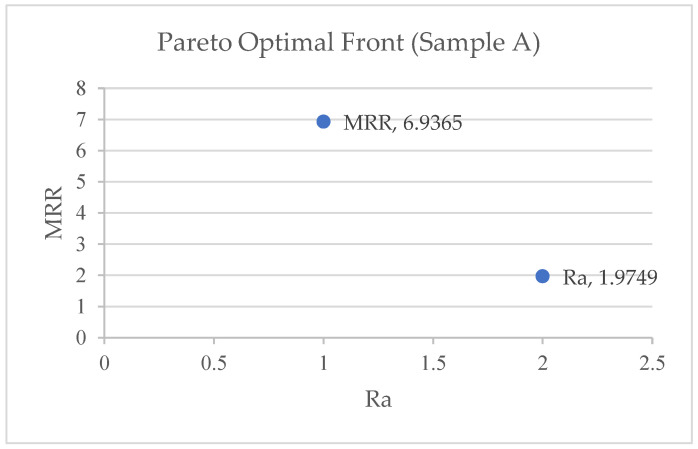
Pareto optimal front for Sample A.

**Figure 4:**
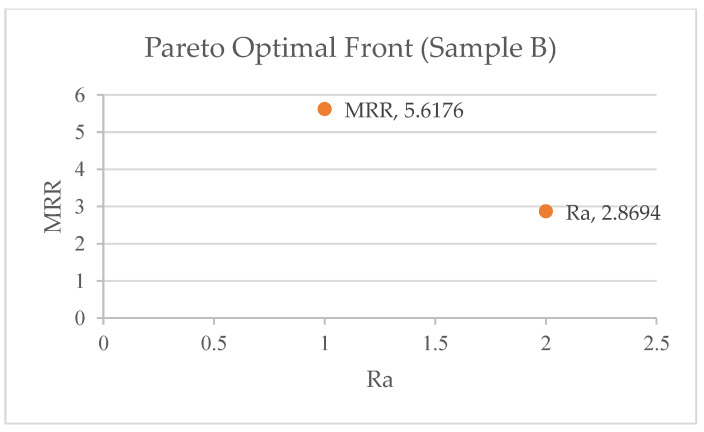
Pareto optimal front for Sample B.

**Figure 5:**
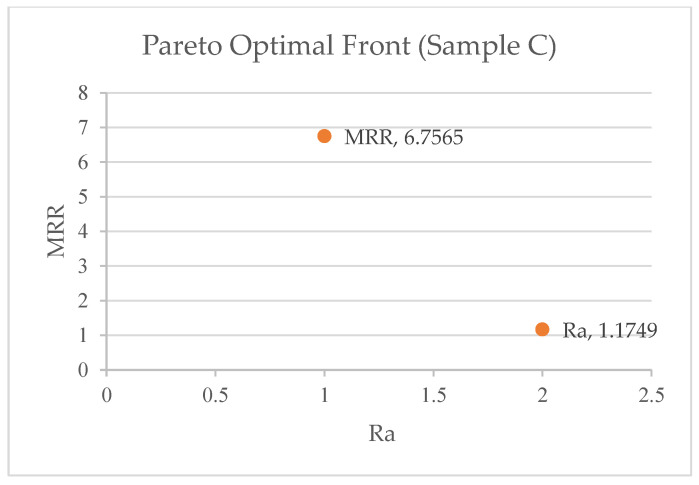
Pareto optimal front for Sample C.

ANOVA was performed using Equations (2)–(7) to create objective functions for the MRR and SR. Table 7 shows the Pareto optimum solutions from a study conducted to improve the surface quality and material removal rate. The study used a Pareto fraction (0.1) and a population size (1000) to change three machining parameters: pulse-active (Ton) time, servo (sv) voltage, and peak current (pc) amplitude. The table has 10 solution sets, with solution 2 being the best. Each solution set is identified by its Ton, servo voltage, peak current, MRR (mm^3^/min), and Ra (μm surface roughness). The two objective functions from ANOVA are MRR and Ra. The table illustrates that the Ton, servo voltage, and peak current vary between solution sets, resulting in varied MRR and SR values. Figure 3, Figure 4 and Figure 5 shows the 10 pareto optimal frontal findings, showing improved convergence. Any solution from Table 7 is suitable depending on product needs, but when the best pareto optimum solution set is picked to achieve superior productivity and quality factors (MRR and roughness), it is found that solution 5 is the best for Samples A and C, whereas solution 4 is the best for Sample B.

In this study, the WEDM process parameters for Al6061-based composites were optimized, resulting in a significant improvement in the Material Removal Rate (MRR). The results show an MRR of 6.9365 mm^3^/min for a 5 mm thickness plate, which is in line with previous studies by Anand et al. [33], who reported an MRR of [7.793 mm^3^/min] aluminum composites. However, the present findings differ from the results of Kumar et al. [34], with an MRR of 11.4 mm^3^/min for the composite material but with different parametric settings for a 12 mm thickness plate. This difference could be due to the differences in the reinforcement type, which may alter the material’s conductivity and response to the WEDM process. Regarding the surface quality, the present study achieved an average surface roughness (Ra) of 1.9749 μm, which compares favorably to the work of Motorcu et al. [35], who reported roughness (Ra) values of 3.384 μm for Al-based composites machined using WEDM. The slight deviation in the results indicate that the optimized parameter settings in the present study such as higher current intensity and pulse-on time resulted in a more stable discharge and smoother cut. The results suggests that the parameter selection, particularly in terms of energy control during the WEDM process, may lead to a better balance between an efficient material removal and the surface finish quality. Such differences highlight the importance of fine-tuning WEDM parameters to achieve a balance between productivity (material removal rate) and precision (surface quality) in machining Al6061-based composites.

NSGA-II is effectively utilized for the multi-objective optimization of WEDM parameters, focusing on the MRR and surface roughness (SR) for Al6061/Mg/MoS_2_ composites. Modrak et al. [36] used NSGA-II, alongside MOPSO, to optimize the kerf width and SR for Al-Mg-MoS_2_ composites, noting that MOPSO requires fewer iterations and less computational time than NSGA-II. However, this study demonstrates a higher degree of precision, with minimal error deviations (e.g., 0.53% for the MRR in Sample A) compared to the ±6% errors reported by Magabe et al. [37] for Ni55.8 Ti alloys. Mithilesh K. et al. [38] also employed NSGA-II for optimizing the MRR and Ra in die-sinking EDM of Ti6Al4V. While this study shares methodologies with these studies, it stands out by achieving exceptionally low error margins and confirming the suitability of NSGA-II for reliable optimization, emphasizing the model’s effectiveness in balancing the MRR and SR.

## 4. Optimization Validation

The final step is to validate and confirm the improvement in the performance characteristic using the optimum level of the process variables obtained using NSGA-II. A confirmation experiment was performed on the optimal solution set 2 and the results of the verification experiment using the optimum procedure variables are shown in Table 8, along with a comparison of the NSGA-II for the optimal and experimental process variable values. According to Table 8, the MRR dropped by 0.47% for sample A and 1.62% for sample C, whereas it increased by 0.53% for sample B. On the other hand, the SR decreased by 174% for sample A and 1.75% for sample B, and increased by 0.89% for sample C.

## 5. Phase Composition Analysis

**Figure 6:**
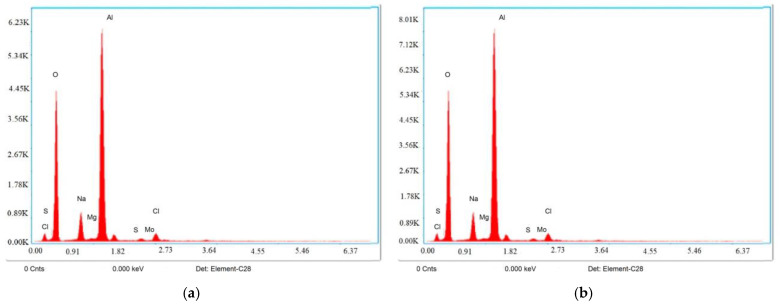
EDX image of machined surface before (**a**) and after (**b**) WEDM.

The Al6061/Mg/MoS_2_ composites’ energy-dispersive X-ray spectroscopy (EDS) measurement prior to machining is displayed in Figure 6a. A clean and unaltered surface is indicated by the EDX spectrum’s prominent aluminum (Al) peaks and weaker levels of oxygen (O), magnesium (Mg), sulfur (S), and molybdenum (Mo). The energy-dispersive X-ray spectroscopy (EDS) study of the Al6061/Mg/MoS_2_ composites following machining is displayed in Figure 6b. The higher oxygen peaks indicate that an oxide layer may have formed as a result of the high temperatures produced during machining. The stability of the other elemental peaks (Al, Mg, S, and Mo) indicates that the composition of the base material has not changed much.

## 6. Limitations and Further Study

This study optimized the WEDM parameters for Al6061/Mg/MoS_2_ composites. The preparation and mixing operations were handled with care to achieve a consistent material structure. However, the precise composition, particularly the distribution of reinforcing elements, has a major impact on machining performance. This is not intended to undermine the validity of the findings, but rather presents an exciting opportunity for further investigation. Future research into the consequences of compositional changes could provide new insights and lead to even greater refinements in machining techniques, hence improving the practical applications of these composites in the industry.

## 7. Conclusions

WEDM effectively processes challenging materials like titanium alloys, aluminum-based MMCs, and Inconel by optimizing parameters to improve the surface roughness (Ra) and Material Removal Rate (MRR). This study advances these techniques, using the Box–Behnken Design together with NSGA-II to achieve an ideal MRR and Ra balance for Al6061/Mg/MoS_2_ composites. The high-accuracy quadratic model developed here (with errors under 2%) reliably predicts WEDM outcomes, setting a foundation for broader applications in machining complex MMCs.

- The NSGA-II optimization method significantly minimizes the deviation between the predicted and experimental values of the Material Removal Rate (MRR) and surface roughness (SR), underscoring its effectiveness and potential impact on WEDM optimization. For Sample A, the predicted MRR from NSGA-II was 6.9365 mm^3^/min, and the SR was 1.9749 µm, while the experimental values were 6.9 mm^3^/min and 1.941 µm, with a deviation of 0.53% for the MRR and 1.74% for the SR.
- Similar minimal deviations were observed for Samples B and C, further validating the effectiveness of the optimization. For Sample B, the MRR deviation was 0.53%, and the SR deviation was 1.75%; for Sample C, the MRR deviation was 1.62%, and the SR deviation was 0.89%.
- The quadratic model used to characterize the non-linear behavior of the MRR and SR was found to be suitable, with high F-values and R^2^ values greater than 0.92. This strong correlation between the predicted and experimental values instills confidence in the accuracy of the NSGA-II method. The analysis revealed that the pulse-active (Ton) time and servo voltage (SV) have a significant impact on the MRR and SR, respectively.
- This finding provides valuable insights into the key factors influencing the optimization results, enhancing our understanding of the process. The NSGA-II optimization approach has proven highly efficient, successfully achieving multiple optimization goals and producing Pareto-optimal solutions.
- This approach effectively balances surface properties and the material removal rate, providing reassurance of its effectiveness. The minimal deviation between the predicted and experimental values, alongside the high precision and accuracy, highlighted the NSGA-II method’s ability to improve WEDM performance compared to conventional methods.
- The findings underscore the value of NSGA-II for WEDM optimization, significantly improving precision and achieving optimal process parameters, which enhance the overall machining process.
- The study provides valuable insights into optimizing WEDM parameters for Al6061/Mg/MoS_2_ composites, although the results may be influenced by the specific composition of the materials used. Future research should consider varying material compositions to validate and expand upon these findings.

## Figures and Tables

**Table 1:** Control variables with levels.

Parameters/Levels	−1 Level	+1 Level
Pulse active (Ton) time (µs)	100	110
Servo (SV) Voltage (v)	30	40
Peak amplitude (PC) of current (A)	30	32

**Table 2:** Box–Behnken design with response values.

Expt. No.	Input Parameters			Responses					
				Sample A		Sample B		Sample C	
	A-Ton (µs)	B-SV (v)	C-PC (A)	MRR mm^3^/min	SR µm	MRR mm^3^/min	SR µm	MRR mm^3^/min	SR µm
1.	110	30	31	7.01	3.652	5.91	4.552	6.91	2.652
2.	105	30	32	7.31	3.740	6.31	4.640	7.21	3.04
3.	105	30	30	7.26	3.754	6.26	4.654	7.16	2.954
4.	100	30	31	7.13	3.831	6.23	4.431	7.33	2.931
5.	100	40	31	7.23	3.773	6.13	4.373	7.33	3.173
6.	105	40	30	7.49	3.724	6.69	4.724	7.29	2.724
7.	105	40	32	7.51	3.706	6.71	4.306	7.51	3.006
8.	110	40	31	7.03	3.640	6.13	4.240	7.03	3.04
9.	110	35	32	7.07	3.611	6.27	4.611	6.87	2.811
10.	110	35	30	7.04	3.630	5.94	4.330	7.04	2.63
11.	100	35	30	7.15	3.823	6.25	4.623	7.35	3.123
12.	100	35	32	7.19	3.800	6.39	4.400	7.29	2.8
13.	105	35	31	7.22	3.744	6.22	4.344	7.42	3.144
14.	105	35	31	7.28	3.751	6.48	4.351	7.28	3.151
15.	105	35	31	7.31	3.736	6.41	4.536	7.01	3.136
16.	105	35	31	7.32	3.753	6.22	4.453	7.32	3.053
17.	105	35	31	7.28	3.748	6.28	4.548	7.28	2.948

**Table 3:** Variance analysis for the MRR and SR models (Samples A, B, and C).

Responses	Sample A			Sample B			Sample C		
	R^2^	Adj. R^2^	Adeq. Precision	R^2^	Adj. R^2^	Adeq. Precision	R^2^	Adj. R^2^	Adeq. Precision
MRR	0.9879	0.9722	26.411	0.9626	0.9146	15.428	0.9731	0.9386	16.976
SR	0.9439	0.8718	12.197	0.9335	0.8481	11.172	0.9205	0.8183	11.366

**Table 4:** Variance analysis and process parameter coefficients for MRR and SR (Sample A).

Source	Model for MRR					Model for SR				
	Quadratic Sum	df	Mean Square	F-Value	*p*-Value	Quadratic Sum	df	Mean Square	F-Value	*p*-Value
Model	0.0659	9	0.0073	63.27	<0.0001	0.3215	9	0.0357	13.09	0.0013
A-Pulse active (Ton) time	0.0602	1	0.0602	519.84	<0.0001	0.0378	1	0.0378	13.85	0.0074
B-Servo Voltage (SV)	0.0022	1	0.0022	19.38	0.0031	0.0378	1	0.0378	13.85	0.0074
C-Peak amplitude of current (PC)	0.0007	1	0.0007	5.91	0.0453	0.0025	1	0.0025	0.8977	0.375
AB	0.0005	1	0.0005	4.57	0.0699	0.0016	1	0.0016	0.5862	0.4689
AC	4.00 × 10^−6^	1	4.00 × 10^−6^	0.0345	0.8578	0	1	0	0.0092	0.9264
BC	4.00 × 10^−6^	1	4.00 × 10^−6^	0.0345	0.8578	0.0002	1	0.0002	0.0824	0.7823
A^2^	0.0015	1	0.0015	12.71	0.0092	0.2247	1	0.2247	82.32	<0.0001
B^2^	0.0001	1	0.0001	0.4977	0.5033	0.0101	1	0.0101	3.7	0.0957
C^2^	0.0006	1	0.0006	4.98	0.0609	0.0159	1	0.0159	5.83	0.0464
Residual	0.0008	7	0.0001			0.0191	7	0.0027		
Lack of Fit	0.0006	3	0.0002	4.63	0.0864	0.013	3	0.0043	2.86	0.1684
Pure Error	0.0002	4	0			0.0061	4	0.0015		
Cor Total	0.0668	16				0.3406	16			

**Table 5:** Variance analysis and process parameter coefficients for MRR and SR (Sample B).

Source	Model for MRR					Model for SR				
	Quadratic Sum	df	Mean Square	F-Value	*p*-Value	Quadratic Sum	df	Mean Square	F-Value	*p*-Value
Model	0.0669	9	0.0074	20.04	0.0003	0.3364	9	0.0374	10.92	0.0023
A-Pulse active (Ton) time	0.0602	1	0.0602	162.17	<0.0001	0.0703	1	0.0703	20.55	0.0027
B-Servo Voltage (SV)	0.0022	1	0.0022	6.05	0.0435	0.0253	1	0.0253	7.4	0.0298
C-Peak amplitude of current (PC)	0.0007	1	0.0007	1.84	0.2166	0.0072	1	0.0072	2.1	0.1902
AB	0.0011	1	0.0011	2.93	0.1305	0.0001	1	0.0001	0.0292	0.8691
AC	0.0001	1	0.0001	0.3879	0.5532	0.003	1	0.003	0.8839	0.3784
BC	0.0001	1	0.0001	0.1724	0.6904	0.0002	1	0.0002	0.0657	0.805
A^2^	0.001	1	0.001	2.62	0.1495	0.1964	1	0.1964	57.4	0.0001
B^2^	1.68 × 10^−7^	1	1.68 × 10^−7^	0.0005	0.9836	0.0334	1	0.0334	9.75	0.0168
C^2^	0.0014	1	0.0014	3.76	0.0938	0.0112	1	0.0112	3.26	0.1138
Residual	0.0026	7	0.0004			0.024	7	0.0034		
Lack of Fit	0.0021	3	0.0007	5.47	0.0671	0.0023	3	0.0008	0.1399	0.931
Pure Error	0.0005	4	0.0001			0.0217	4	0.0054		
Cor Total	0.0695	16				0.3604	16			

**Table 6:** Variance analysis and process parameter coefficients for MRR and SR (Sample C).

Source	Model for MRR					Model for SR				
	Quadratic Sum	df	Mean Square	F-Value	*p*-Value	Quadratic Sum	df	Mean Square	F-Value	*p*-Value
Model	0.0586	9	0.0065	28.18	0.0001	0.3291	9	0.0366	9.01	0.0042
A-Pulse active (Ton) time	0.0535	1	0.0535	231.2	<0.0001	0.0475	1	0.0475	11.71	0.0111
B-Servo Voltage (SV)	0.0022	1	0.0022	9.71	0.017	0.0475	1	0.0475	11.71	0.0111
C-Peak amplitude of current (PC)	0.0007	1	0.0007	2.96	0.129	0.0053	1	0.0053	1.32	0.2891
AB	0.0002	1	0.0002	0.7308	0.4209	0	1	0	0.0109	0.9196
AC	0.0001	1	0.0001	0.2768	0.6151	0.0051	1	0.0051	1.27	0.2977
BC	0.0001	1	0.0001	0.6227	0.4559	0.0027	1	0.0027	0.6576	0.4441
A^2^	0.001	1	0.001	4.21	0.0794	0.1995	1	0.1995	49.14	0.0002
B^2^	0.0004	1	0.0004	1.89	0.2111	0.0164	1	0.0164	4.03	0.0847
C^2^	0.0003	1	0.0003	1.22	0.3051	0.0142	1	0.0142	3.51	0.1032
Residual	0.0016	7	0.0002			0.0284	7	0.0041		
Lack of Fit	0.001	3	0.0003	2.1	0.2434	0.0051	3	0.0017	0.2942	0.8287
Pure Error	0.0006	4	0.0002			0.0233	4	0.0058		
Cor Total	0.0603	16				0.3575	16			

**Table 7:** Pareto optimum frontal solutions.

S. No	Pareto Optimal Solutions								
	Ton	Servo Voltage	Peak Current	Sample A		Sample B		Sample C	
				MRR	Ra	MRR	Ra	MRR	Ra
1	110	29	31	6.5149	3.1261	4.9149	4.0261	5.9149	2.1261
2	110	27	32	5.7974	2.073	4.5974	2.973	5.4974	1.373
3	110	27	30	5.8792	2.4358	4.6292	3.3358	5.5292	1.6358
4	110	30	31	6.8976	2.3621	5.6176	2.8694	6.7176	1.4621
5	110	30	31	6.9365	1.9749	5.5565	2.9621	6.7565	1.1749
6	110	28	30	6.3498	3.7201	5.5298	4.7201	6.1298	2.7201
7	110	27	32	5.9567	2.5198	5.1567	3.1198	5.9567	1.8198
8	110	28	31	6.4243	3.3902	5.0443	3.9902	5.9443	2.7902
9	110	27	32	5.8669	2.2694	4.6269	2.9749	5.2269	1.6694
10	110	29	30	6.4724	3.1427	4.9024	3.8427	6.0024	2.1427

**Table 8:** The NSGA verification experiment’s results (Samples A, B, and C).

Input Parameters				Responses					
Ton	Servo Voltage	Peak Current		Sample A		Sample B		Sample C	
				MRR	SR	MRR	SR	MRR	SR
110	30	31	NSGA-II	6.9365	1.9749	5.6176	2.8694	6.7565	1.1749
			Experiment	6.9	1.941	5.71	2.844	6.71	1.1823
			% Deviation	0.53%	1.74%	0.53%	1.75%	1.62%	0.89%

## Data Availability

The original contributions presented in the study are included in the article, further inquiries can be directed to the corresponding author.
